# Advancing photovoltaics with Cs_2_NaInI_6_-based perovskites: a simulation study on ETL optimization

**DOI:** 10.1039/d5ra05885f

**Published:** 2025-10-13

**Authors:** Md. Ferdous Rahman, Md. Azizur Rahman, Mutasem Z. Bani-Fwaz, Md. Faruk Hossain, Nacer Badi, Aijaz Rasool Chaudhry, Ahmad Irfan

**Affiliations:** a Advanced Energy Materials and Solar Cell Research Laboratory, Department of Electrical and Electronic Engineering, Begum Rokeya University Rangpur 5400 Bangladesh ferdousapee@gmail.com; b Department of Chemistry, College of Science, King Khalid University P. O. Box 9004 Abha 61413 Saudi Arabia; c Department of Physics, Rajshahi University of Engineering & Technology Rajshahi 6204 Bangladesh; d Department of Physics, Faculty of Science, University of Tabuk Tabuk 71491 Saudi Arabia; e Department of Physics, College of Science, University of Bisha P. O. Box 551 Bisha 61922 Saudi Arabia

## Abstract

Developing reliable, energy-efficient, and eco-friendly photovoltaic materials is crucial for advancing next-generation solar technologies. Among lead-free options, double perovskites such as Cs_2_NaInI_6_ show strong potential due to their direct bandgap (∼1.6 eV), excellent light absorption, high carrier mobility, and environmental durability. The efficiency of Cs_2_NaInI_6_-based perovskite solar cells (PSCs), however, is strongly influenced by the electron transport layer (ETL). In this work, Solar Cell Capacitance Simulator in One Dimension (SCAPS-1D) simulations were employed to analyze ITO/ETL/Cs_2_NaInI_6_/Au structures using WS_2_, SnS_2_, In_2_S_3_, and IGZO as ETLs. Critical factors—absorber thickness, defect density, doping levels, interface traps, and temperature—were systematically tuned to assess their effects on power conversion efficiency (PCE), open-circuit voltage (*V*_OC_), short-circuit current density (*J*_SC_), and fill factor (FF). Among the tested ETLs, WS_2_ delivered the best performance with a PCE of 22.63%, *V*_OC_ of 1.189 V, *J*_SC_ of 21.406 mA cm^−2^, and FF of 88.92%, attributed to favorable band alignment, high mobility, and reduced recombination. Additionally, Cs_2_NaInI_6_ demonstrated promising thermal and defect stability, emphasizing its viability for real-world applications. Overall, this study underscores the critical role of ETL engineering and provides a simulation-guided approach for designing efficient lead-free perovskite solar cells (PSCs).

## Abstract 2

Developing reliable, energy-efficient, and eco-friendly photovoltaic materials is crucial for advancing next-generation solar technologies.

## Graphical abstract

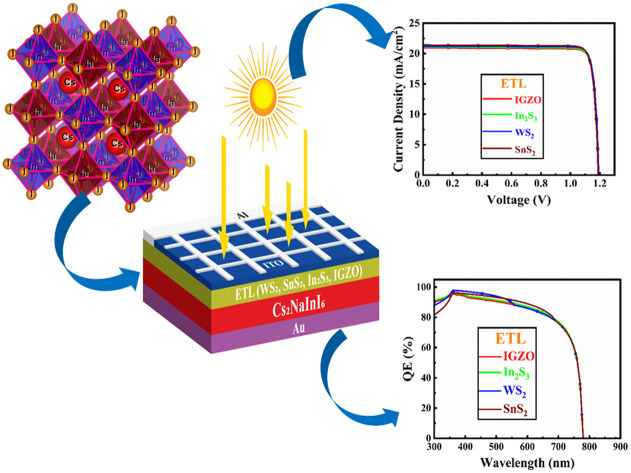

## 1. Introduction

Growing global demand for sustainable energy drives photovoltaic (PV) innovation. Achieving high solar conversion efficiency remains challenging due to intrinsic and extrinsic losses from material imperfections, interfacial recombination, and energy mismatches, all closely linked to the properties of photoactive materials and the design of device architectures.^1,2^ First-generation silicon-based photovoltaics dominate the market for their efficiency and reliability, but energy-intensive wafer fabrication raises costs, limiting broader adoption despite their commercial success. Second-generation thin-film solar cells use fewer materials and lower temperatures but face sustainability concerns from Cd toxicity and scarce indium reserves.^3,4^ To overcome these constraints, third-generation solar cell technologies—notably organic photovoltaics, quantum dot solar cells, and perovskite-based devices—have emerged as attractive alternatives.^5^ Among them, halide perovskites have stimulated widespread academic attention owing to their exceptional power conversion efficiencies (PCEs),^6^ adjustable bandgaps, strong optical absorption capabilities, and extended charge carrier diffusion lengths. Lead perovskites suffer from instability and toxicity, while double perovskites (A_2_BB'X_6_) provide stable, eco-friendly alternatives for sustainable photovoltaic absorber development.^7–10^ Double perovskites (A_2_BB'X_6_), derived from the ABX_3_ framework with ordered cation substitution, enable tunable structural, electronic, and optical properties for photovoltaics.^11^ Non-lead variants like Cs_2_AgBiBr_6_, Cs_2_TiBr_6_, and Cs_2_SnI_6_ show promise as eco-friendly alternatives^12–14^ but face challenges including wide/indirect bandgaps, poor charge transport, and limited defect tolerance, restricting solar cell efficiencies below 2%.^15,16^

Recently, Cs_2_NaInI_6_ has become recognized as a compelling lead-free double perovskite absorber material, distinguished by its desirable optoelectronic and structural properties. It reveals a direct bandgap around 1.6 eV, closely matching the optimal range for single-junction solar cells as defined by the Shockley–Queisser limit. Additionally, it boasts a strong absorption coefficient (∼10^6^ cm^−1^), excellent charge carrier mobility, and exceptional thermal and moisture stability.^17,18^ The material's Goldschmidt tolerance factor (∼0.88) ensures a stable cubic structure, and its resistance to oxidation further enhances device longevity under operational stress.^19^ These attributes make Cs_2_NaInI_6_ a highly promising platform for stable, lead-free, and efficient solar energy harvesting. While the photoactive properties of Cs_2_NaInI_6_ are well documented, the comprehensive effectiveness of a solar cell strongly relies on its interfacial layers, especially the ETL. The ETL serves an essential function in facilitating charge extraction, suppressing carrier recombination, and maintaining appropriate energy band orientation with the active layer.^20–22^ Hence, the judicious selection and optimization of the ETL are crucial for achieving superior performance in PSCs.

This research provides a thorough simulation-driven analysis of the photovoltaic behavior of Cs_2_NaInI_6_-based PSCs employing four different ETL materials WS_2_, SnS_2_, In_2_S_3_, and IGZO within a planar heterojunction configuration of ITO/ETL/Cs_2_NaInI_6_/Au. The choice of ETL materials (WS_2_, SnS_2_, In_2_S_3_, and IGZO) was motivated by their favorable electronic and physical compatibility with Cs_2_NaInI_6_. WS_2_ and SnS_2_ possess high electron mobilities and tunable band alignments that form small positive conduction band offsets with Cs_2_NaInI_6_, which helps suppress interfacial recombination while enabling efficient charge extraction.^23,24^ In_2_S_3_, a widely used n-type semiconductor, has been investigated for its benign processing conditions and ability to form relatively stable interfaces with halide perovskites.^25^ IGZO, an amorphous oxide semiconductor with good transparency and high mobility, is compatible with large-area, low-temperature deposition methods, making it attractive for scalable photovoltaic applications.^26,27^ Thus, these four candidates were selected to comparatively evaluate their suitability as ETLs for Cs_2_NaInI_6_ absorbers. Simulations were performed applying SCAPS-1D, a widely validated numerical tool for assessing the optoelectronic characteristics of multilayer solar cells. The study systematically explores the role of absorber thickness, defect density (both in the bulk and at interfaces), shallow donor/acceptor densities, and transport layer properties on device performance metrics. Notably, WS_2_ demonstrated the best performance among the evaluated ETLs, followed by SnS_2_, In_2_S_3_, and IGZO, respectively. By highlighting the synergistic role of ETL material selection and device engineering, this work establishes a framework for optimizing Cs_2_NaInI_6_-based lead-free perovskite solar cells. The results offer critical insights into interface-dependent performance enhancement and provide a roadmap for future experimental realization of efficient, stable, and environmentally benign perovskite photovoltaics.

## 2. Methodology

### 2.1. Simulation utilizing SCAPS-1D

This study employs numerical modeling and simulation of ITO/ETL (WS_2_, SnS_2_, In_2_S_3_, and IGZO)/Cs_2_NaInI_6_/Au PSCs applying the SCAPS-1D. SCAPS-1D is a widely utilized simulation tool in photovoltaic research, known for its ability to accurately replicate experimental performance trends.^1,2^ The software enables detailed device modeling by supporting up to seven semiconductor layers, six interface regions, and two electrical contacts.^28^ It computes key photovoltaic output parameters through the computational approach by solving the core equations of semiconductor physics. These include Poisson's equation, the electron and hole continuity equations, and the current density equations, which collectively describe the electrostatic potential and carrier transport dynamics within the device structure.^29^ The governing equations and their boundary conditions form the basis for simulating the steady-state behavior of the PSC and are elaborated comprehensively in the referenced literature^29,30^ and mentioned below.1
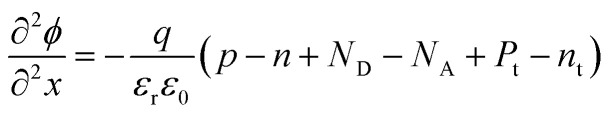
where, *ε* represents the dielectric constant, *n* denotes the electron concentration, *p* stands for the hole concentration, *q* corresponds to the elementary charge, *N*_D_ refers to the donor concentration, and *N*_A_ designates the acceptor concentration.2
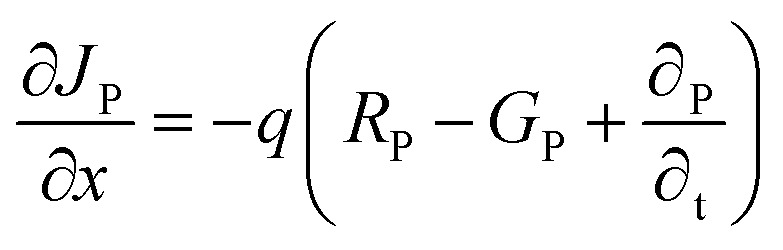
3
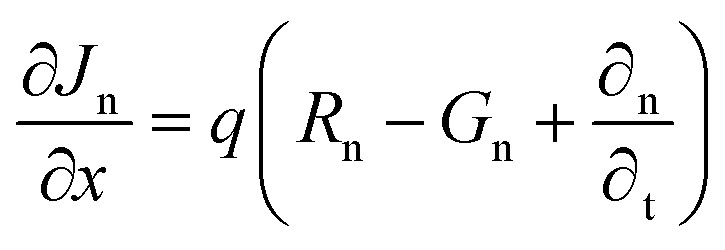
Here, *J*_P_ and *J*_n_ represent the current densities of holes and electrons, respectively, while *R*_P_ and *G*_P_ denote the rates of hole recombination and generation. Furthermore, *G*_n_ indicates the electron generation rate, and *R*_n_ corresponds to the electron recombination rate.4
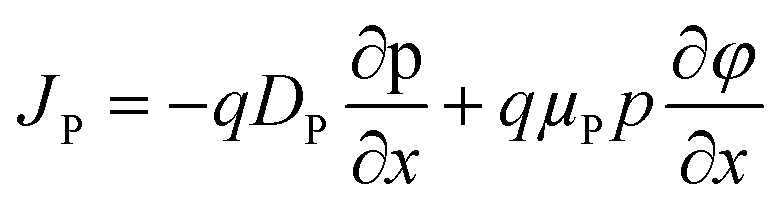
5
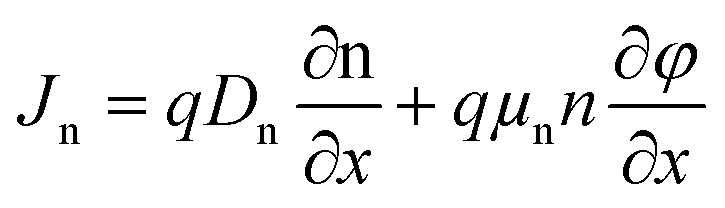
Here, *D*_P_ and *D*_n_ denote the diffusion coefficients for holes and electrons, respectively, while *μ*_P_ and *μ*_n_ represent the mobilities of holes and electrons.

### 2.2. The Cs_2_NaInI_6_-based PSC structure

**Fig. 1:**
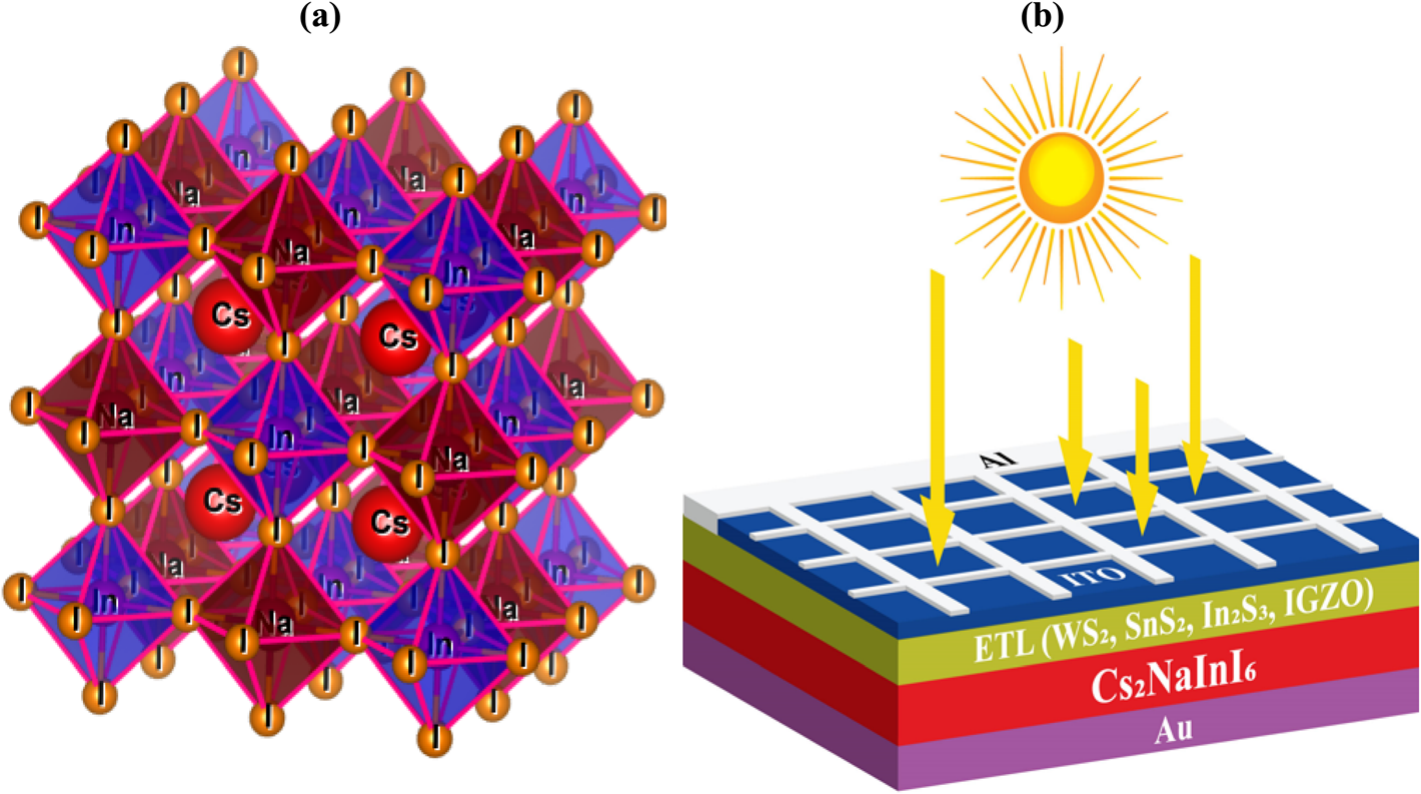
(a) Crystal structure of the Cs_2_NaInI_6_, and (b) device architecture of the Cs_2_NaInI_6_-based PSC.

The absorber crystal structure has shown in the Fig. 1(a). On the other hand, the proposed planar heterojunction PSC architecture, ITO/ETL (WS_2_, SnS_2_, In_2_S_3_, IGZO)/Cs_2_NaInI_6_/Au, illustrated in Fig. 1(b), Cs_2_NaInI_6_ is employed as the key light-absorbing layer in the device architecture. This material plays a central role in enhancing solar cell performance because of its optimal bandgap, which is well-suited for photovoltaic applications, and its strong optical absorption coefficient, enabling efficient harvesting of incident photons. The aluminum (Al) grid shown in the schematic represents the front contact metallization, which improves charge extraction by providing low-resistance pathways and thereby minimizes series resistance losses. Indium-doped tin oxide (ITO) is utilized as the transparent conducting oxide (TCO) at the front contact, allowing both high optical transparency and good electrical conductivity. Finally, gold (Au) functions as the rear metal electrode of the perovskite solar cell, with a work function of approximately 5.1 eV, ensuring efficient hole collection and stable back contact formation.^20^ With the intention of analyzing the effect of ETLs on device output, four materials including WS_2_, SnS_2_, In_2_S_3_, and indium gallium zinc oxide (IGZO) were systematically investigated. The material parameters and layer-specific properties used in the simulations are detailed in Tables 1 and 2. All computational analyses were accomplished using standard AM1.5 G illumination conditions with an incident power density of 1000 mW cm^−2^ and an ambient operating temperature of 300 K, confirming consistency with real-world solar cell test environments.

**Table 1:** The parameters used for the proposed device structure

Parameters	ITO^20^	IGZO^20,31^	In_2_S_3_ (ref. 32)	WS_2_ (ref. 20)	SnS_2_ (ref. 33)	Cs_2_NaInI_6_ (ref. 34)
Thickness (nm)	30	30	50	100	100	1200
Band gap, *E*_g_ (eV)	3.5	3.05	2.82	1.80	2.24	1.6
Electron affinity, *χ* (eV)	4.0	4.16	4.50	3.95	4.24	4.379
Dielectric permittivity (relative), *ε*_r_	9.0	10	13.50	13.60	10.0	3.5
Effective density of states in conduction band, *N*_C_ (cm^−3^)	2.2 × 10^18^	5.0 × 10^18^	2.2 × 10^17^	1.0 × 10^18^	2.2 × 10^18^	3.16 × 10^18^
Effective density of states in valence band, *N*_V_ (cm^−3^)	1.8 × 10^19^	5.0 × 10^18^	1.8 × 10^19^	2.4 × 10^19^	1.80 × 10^19^	1.71 × 10^19^
Hole mobility, *μ*_h_ (cm^2^ V^−1^ s ^−1^)	10	10	25	100	50	50
Electron mobility, *μ*_n_ (cm^2^ V^−1^ s ^−1^)	20	15	100	100	50	50
Electron thermal velocity (cm s^−1^)	1 × 10^7^	1 × 10^7^	1 × 10^7^	1 × 10^7^	1 × 10^7^	1 × 10^7^
Hole thermal velocity (cm s^−1^)	1 × 10^7^	1 × 10^7^	1 × 10^7^	1 × 10^7^	1 × 10^7^	1 × 10^7^
Shallow uniform donor density, *N*_D_ (cm^−3^)	1 × 10^18^	1 × 10^19^	1 × 10^16^	1 × 10^19^	1 × 10^18^	0
Shallow uniform acceptor density, *N*_A_ (cm^−3^)	0	0	0	0	0	1 × 10^18^
Defect density, *N*_t_ (cm^−3^)	1 × 10^16^	1 × 10^14^	1 × 10^15^	1 × 10^16^	1 × 10^15^	1 × 10^14^

**Table 2:** The interface parameters applied in the Cs_2_NaInI_6_-based PSCs

Parameters	Cs_2_NaInI_6_/IGZO	Cs_2_NaInI_6_/In_2_S_3_	Cs_2_NaInI_6_/WS_2_	Cs_2_NaInI_6_/SnS_2_
Total defect density (cm^−2^)	10^13^	10^13^	10^13^	10^13^

## 3. Results and discussion

### 3.1. Energy band diagram

**Fig. 2:**
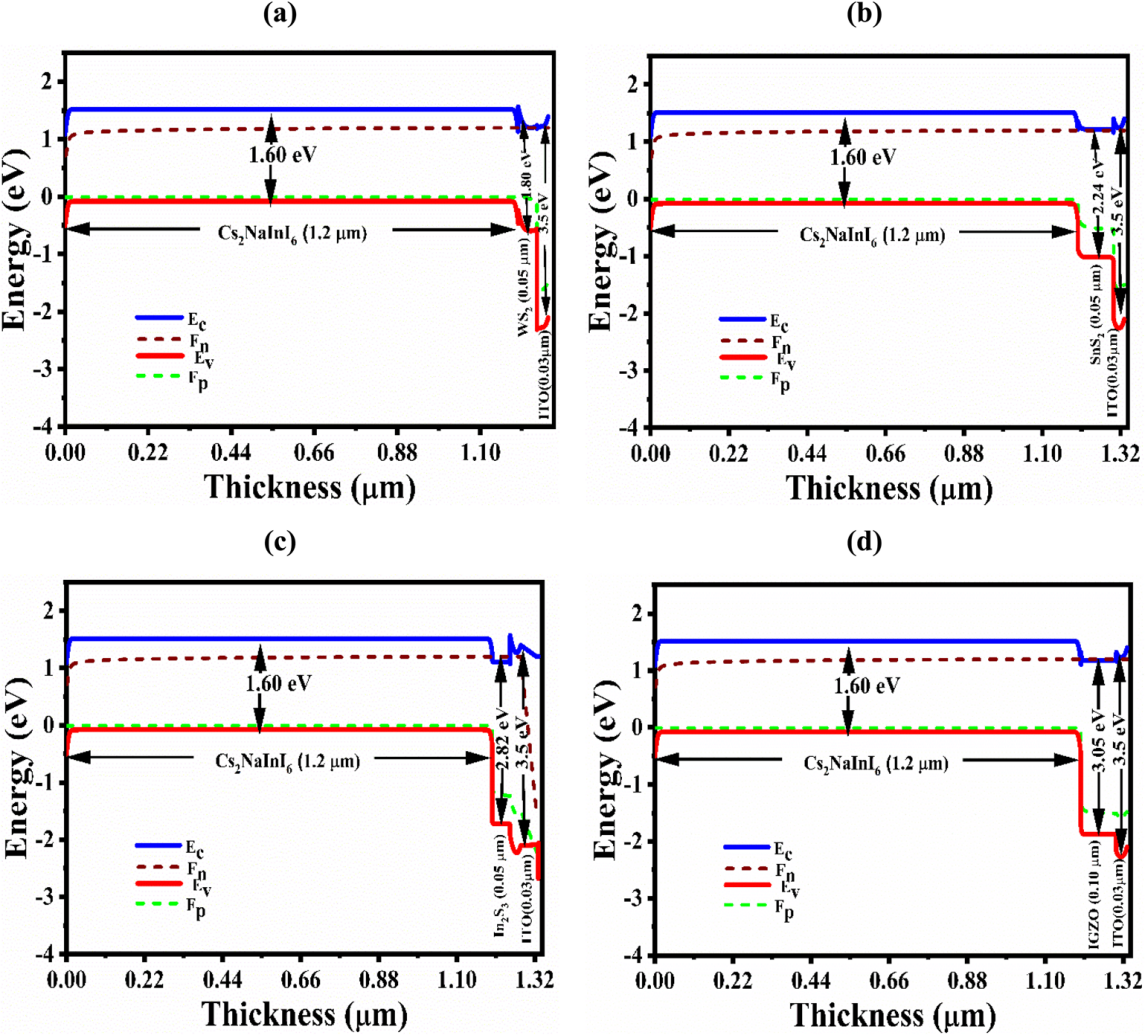
The energy band diagrams of Cs_2_NaInI_6_-based solar cell devices having (a) WS_2_, (b) SnS_2_, (c) In_2_S_3_, and (d) IGZO ETL.

Fig. 2(a)–(d) reveals the band diagrams of the four optimized Cs_2_NaInI_6_-based PSCs. Band alignment discontinuities arise at the interfaces of heterostructures formed by semiconductors with different electronic properties. These interfacial discontinuities are characterized by the conduction band offset (CBO) and valence band offset (VBO), which significantly influence charge carrier dynamics at the absorber/ETL interface.^35,36^ A small positive CBO, or ‘spike’ (∼0–0.3 eV), is generally favorable because it prevents hole back-injection while still enabling efficient electron extraction. In contrast, a large negative CBO, or ‘cliff,’ enhances interfacial recombination and degrades performance. In this study, WS_2_, SnS_2_, and IGZO form small positive spikes with Cs_2_NaInI_6_ due to their slightly lower electron affinities, whereas In_2_S_3_ forms a negative cliff because of its larger electron affinity relative to the absorber. These results are consistent with the values in Table 1 and the band alignments in Fig. 2, underscoring the importance of achieving a modest spike for optimal photovoltaic operation.

As demonstrated in Fig. 2(a)–(d), the quasi-Fermi levels for electrons (*F*_n_) and holes (*F*_P_) appropriately align near the conduction band minimum (*E*_C_) and valence band maximum (*E*_V_), respectively, indicating effective charge separation and transport under illumination. Across all ETLs, *F*_n_ and *E*_C_, while *F*_P_ aligns with *E*_V_ demonstrate a consistent and harmonious affiliation. The band gap of Cs_2_NaInI_6_ is identified as 1.60 eV, with varying band alignments observed across the different ETLs.

### 3.2. Impact of thickness changes of absorber and ETL layer

**Fig. 3:**
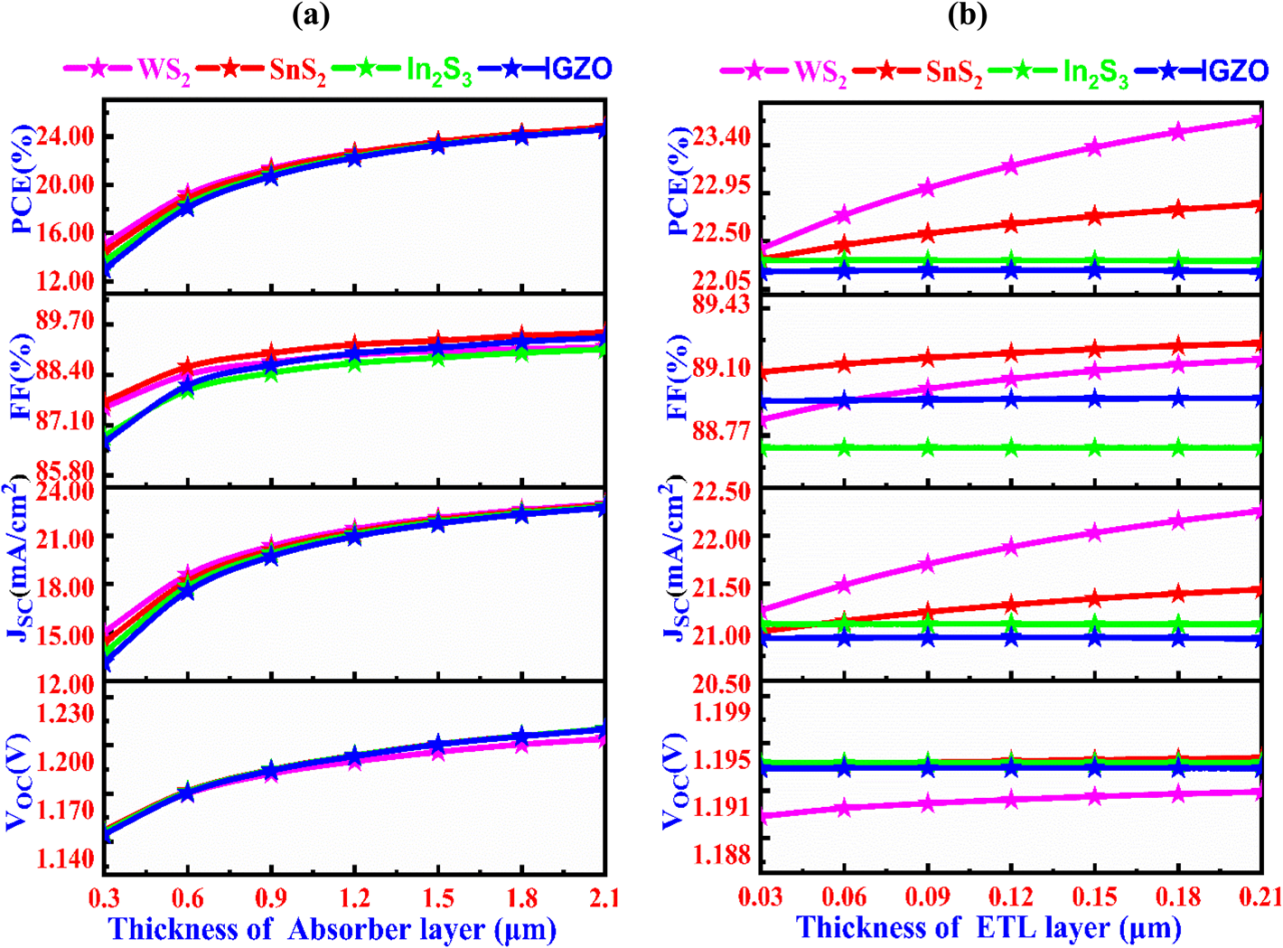
Changes in solar cell characteristics with the variation of (a) Cs_2_NaInI_6_ double perovskite absorber, and (b) ETL thicknesses.

Fig. 3(a) presents the influence of varying the Cs_2_NaInI_6_ double perovskite active layer thickness, varied from 0.3 to 2.1 μm, in determining photovoltaic output of solar cells incorporating different ETL configurations each with a fixed thickness of 100 nm. In the ITO/ETL/Cs_2_NaInI_6_/Au architecture, increasing the absorber thickness leads to marked enhancements in key device parameters, primarily as a result of enhanced light absorption and increased efficiency in generating charge carriers. The enhanced photon absorption with greater thickness leads to higher *J*_SC_ values, while *V*_OC_ and FF improve due to reduced recombination and optimized charge extraction. However, beyond a certain thickness, the performance starts to stabilize, suggesting a suitable absorber thickness that achieves a balance between effective light absorption and optimal charge transport.^37–39^ For the WS_2_ ETL, the device demonstrates a notable improvement in performance metrics with increasing absorber thickness. The *V*_OC_ reveals a gradual increase from 1.147 V to 1.204 V, whereas the *J*_SC_ demonstrates a significant enhancement from 15.02 mA cm^−2^ to 22.95 mA cm^−2^. Simultaneously, the FF enhances moderately from 87.55% to 89.12%, culminating in a substantial enhancement in the PCE from 15.08% to 24.62%. An analogous behavior is seen for the SnS_2_ ETL, where *V*_OC_ increases from 1.15 V to 1.21 V, and *J*_SC_ exhibits a considerable rise from 14.36 mA cm^−2^ to 22.88 mA cm^−2^. The FF improves slightly yet consistently, ranging from 87.70% to 89.49%, while the PCE shows a remarkable gain from 14.45% to 24.77%. In the case of the In_2_S_3_ ETL, the photovoltaic parameters also display favorable progression. The *V*_OC_ increases modestly from 1.15 V to 1.21 V, whereas the *J*_SC_ improves significantly from 13.73 mA cm^−2^ to 22.80 mA cm^−2^, FF rises from 86.82% to 89.06%, and the PCE enhances significantly from 13.67% to 24.57%. Lastly, for the IGZO ETL, the device shows consistent performance enhancement across all metrics. The *V*_OC_ gradually rises from 1.145 V to 1.209 V, while the *J*_SC_ rises appreciably from 13.12 mA cm^−2^ to 22.73 mA cm^−2^, FF improves from 86.67% to 89.36%, and the PCE increases impressively from 13.02% to 24.57%. These improvements are ascribed to enhanced photon capture and more efficient generation of charge carriers with increased thickness.^40^ The optimized absorber thickness for all ETL materials is found to be 1.20 μm, with the WS_2_ ETL showing the best comprehensive performance. This demonstrates the significance of thickness optimization in Cs_2_NaInI_6_-based solar cells, where balancing absorption and charge transport properties maximizes both efficiency and stability.

The Fig. 3(b) demonstrates the role of layer thickness of the ETL on the photovoltaic output of PSCs with absorber thickness of 1.20 μm. As the ETL thickness increases, the PCE rises, with WS_2_ showing the most notable improvement, reaching around 23.40%, while IGZO experiences the smallest gain, reaching about 22.50%. A similar pattern is seen in the FF, where WS_2_ achieves the highest value of 89.10%, while IGZO shows the least change. The *J*_SC_ remains relatively constant for all materials, with slight increases as the ETL thickness grows, and WS_2_ consistently performs better than the others. The *V*_OC_ also shows a modest increase across all materials, with WS_2_ maintaining the highest value near 1.195 V. In conclusion, WS_2_ stands out as the most efficient material in terms of PCE, FF, *J*_SC_, and *V*_OC_, making it the best performer for this ETL setup.

### 3.3. The consequences of variations in the ETL defect density

**Fig. 4:**
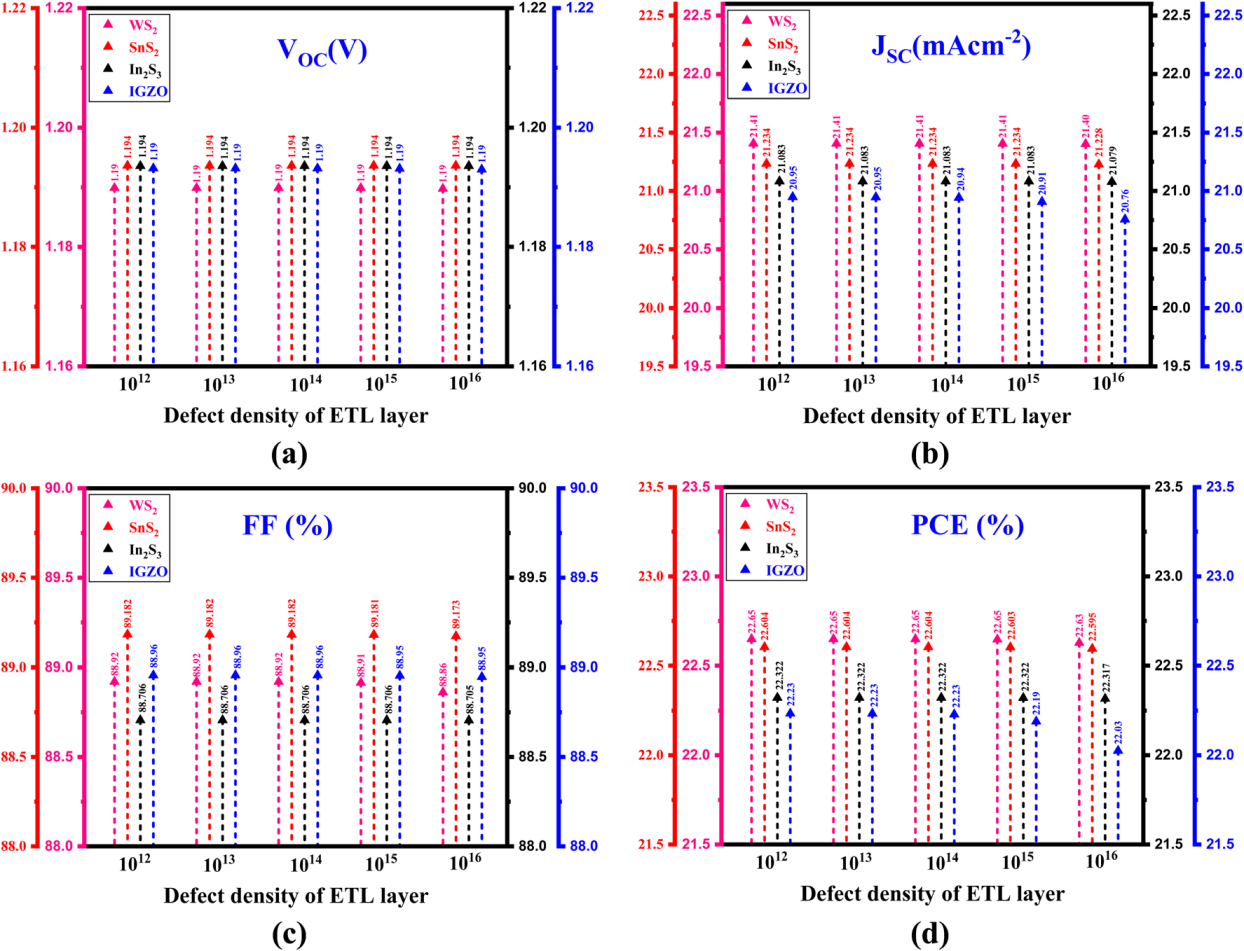
Role of changing ETL defect density on the performance parameter, (a) *V*_OC_, (b) *J*_SC_, (c) FF, and (d) PCE of PSC with different ETL.

Fig. 4 demonstrates the role of changes in the ETL defect density affect the output of different PSC configurations. In the ITO/ETL (WS_2_, SnS_2_, In_2_S_3_ and IGZO)/Cs_2_NaInI_6_/Au structure (Fig. 4(a)), increasing the ETL defect density (Nt) from 10^12^ cm^−3^ to 10^16^ cm^−3^ causes no substantial changes in the *V*_OC_, which remains fixed at 1.19 V for WS_2_ and IGZO, and at 1.194 V for SnS_2_ and In_2_S_3_. This suggests that variations in the defect density do not notably affect the voltage behavior for these materials within this range. In Fig. 4(b), the *J*_SC_ remains stable as far as the defect density of Nt = 10^15^ cm^−3^.

However, exceeding this value, a minor reduction in *J*_SC_ is noticed for all ETL materials, implying that higher defect densities reduce current density, likely due to increased recombination or scattering at the defect sites, which affects charge transport. Fig. 4(c) shows that the FF is constant for In_2_S_3_ and IGZO across all defect densities, indicating that these materials maintain efficient charge extraction despite the defect density variations.^41^ For WS_2_ and SnS_2_, the FF remains steady up to Nt = 10^14^ cm^−3^, but increases in defect density beyond this led to a slight reduction in FF—from 88.92% to 88.87% for WS_2_ and from 88.182% to 88.173% for SnS_2_. This reduction suggests that higher defect densities in these materials slightly impair charge collection efficiency, possibly due to increased recombination or the formation of trap states that hinder charge extraction. Finally, Fig. 4(d) shows that the PCE declines as the defect density of the ETL rises, a trend consistent with the drops in *J*_SC_ and FF. This decrease in PCE highlights the negative role of increased defect density on the overall output of the PSC due to the combined effects of lower current density and less efficient charge collection.

Notably, the impact of ETL defect density on device performance is much more significant for IGZO compared to WS_2_, SnS_2_, and In_2_S_3_. This distinction arises from the amorphous nature of IGZO, which inherently exhibits a higher density of localized sub-gap states.^27^ Defects in IGZO act as efficient recombination centers, thereby intensifying non-radiative losses and limiting charge extraction.^27^ In contrast, crystalline ETLs such as WS_2_, SnS_2_, and In_2_S_3_ are structurally more ordered and display stronger defect tolerance, allowing their photovoltaic behavior to remain relatively stable under similar defect density variations. Consequently, IGZO-based devices show stronger performance degradation with increasing defect density, consistent with the trends observed in Fig. 4.

### 3.4. The result of changes in the ETL shallow donor density

**Fig. 5:**
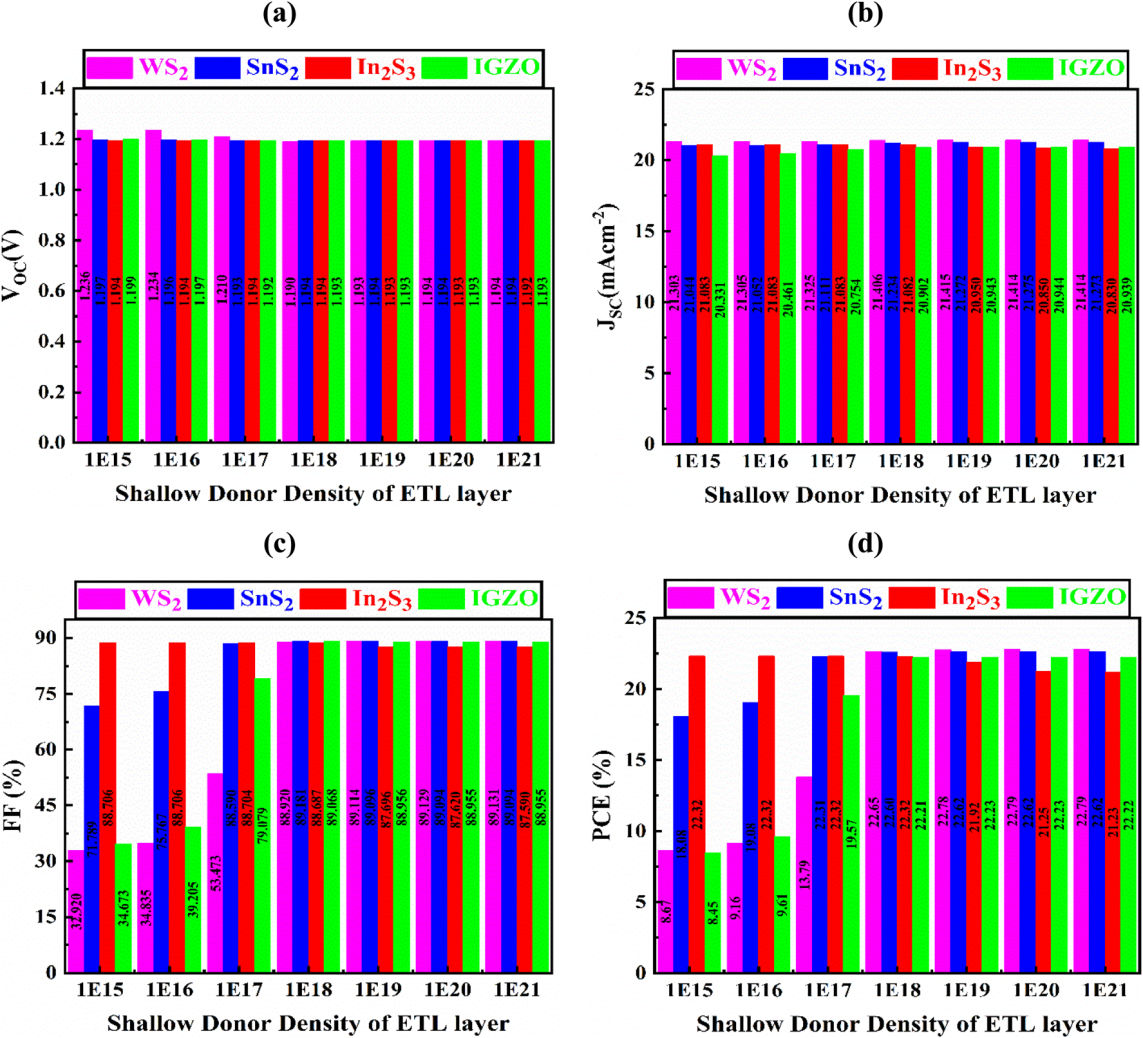
The role of varying ETL shallow donor density on the output, (a) *V*_OC_, (b) *J*_SC_, (c) FF, and (d) PCE of PSCs with various ETL configurations.

Fig. 5(a–d) comprehensively demonstrates the role of varying shallow donor densities (*N*_D_) in the ETLs spanning from 10^15^ to 10^21^ cm^−3^ (ref. 42 and 43) on the photovoltaic operation of PSCs employing various ETL materials. As the shallow donor density increases, a progressive decline in *V*_OC_ is consistently observed across all configurations. The observed decrease is largely caused by the enhanced recombination of charge carriers, as donor-like defect states in the ETL serve as non-radiative recombination centers, thereby reducing the quasi-Fermi level separation and lowering the achievable *V*_OC_.^44^ Specifically, for WS_2_, *V*_OC_ declines markedly from 1.236 V to 1.194 V; for SnS_2_, a subtle lessening from 1.197 V to 1.194 V is detected; In_2_S_3_ exhibits a minor drop from 1.194 V down to 1.192 V; and for IGZO, *V*_OC_ decreases from 1.199 V to 1.193 V. These reductions, while varying in magnitude, underscore the sensitivity of voltage output to defect-induced recombination processes.

In contrast, the *J*_SC_ shows material-specific behavior in response to increased donor density. For WS_2_, a slight but consistent improvement is seen, with *J*_SC_ rising from 21.303 mA cm^−2^ up to 21.414 mA cm^−2^. Similarly, SnS_2_ reveals a moderate increase from 21.044 mA cm^−2^ to 21.273 mA cm^−2^, and IGZO improves from 20.331 mA cm^−2^ to 20.939 mA cm^−2^, suggesting enhanced charge collection possibly due to increased carrier concentration. However, In_2_S_3_ deviates from this trend, exhibiting a marginal decline in *J*_SC_ from 21.083 mA cm^−2^ to 20.830 mA cm^−2^, likely due to a rise in recombination losses outweighing the benefits of elevated carrier concentration. These variations reflect the influence of shallow donor defects on charge transport, with some materials benefiting from increased current density while others show a reduction due to higher defect-related recombination.^45^

The FF reveals substantial changes for various ETL material: for WS_2_, FF increases from 32.92% to 89.13%; for SnS_2_, FF rises from 71.79% to 89.09%; for In_2_S_3_, FF decreases from 88.71% to 87.59%; and for IGZO, FF improves from 34.67% to 88.96%. The increase in FF for most ETL materials indicates better charge extraction efficiency, particularly for WS_2_ and IGZO, which see substantial improvements. Regarding PCE, WS_2_ shows a remarkable increase from 8.87% to 22.79%; SnS_2_ increases from 18.08% to 22.62%; In_2_S_3_ experiences a slight decrease from 22.32% to 21.23%; and IGZO rises from 8.45% to 22.22%. The optimized values for shallow donor defect density are found to be 10^19^ cm^−3^ for WS_2_, 10^18^ cm^−3^ for SnS_2_, 10^16^ cm^−3^ for In_2_S_3_, and 10^19^ cm^−3^ for IGZO. The analysis reveals highlight the critical role of shallow donor defects in the ETL layer, demonstrating that their density significantly influences the optimizing charge transport, solar cell performance, minimizing recombination, and ultimately enhancing the PCE. Furthermore, it is observed in Fig. 5 that the variation of ETL shallow donor density primarily affects the FF, with the effect being most pronounced for WS_2_ and IGZO, while relatively insignificant for In_2_S_3_. This trend can be understood in terms of conductivity enhancement and recombination dynamics. For WS_2_ and IGZO, increased shallow donor density significantly alters carrier transport by improving electron conductivity, but at the same time it can intensify interfacial recombination processes, leading to notable FF fluctuations. In contrast, In_2_S_3_ exhibits more stable performance because its moderate electron affinity^46^ and band alignment with Cs_2_NaInI_6_ result in less sensitivity to donor density changes. Consequently, the FF of In_2_S_3_-based devices remains comparatively stable under varying shallow donor densities, explaining the weaker impact observed in Fig. 5.

Based on the comparative analysis in Sections 3.1–3.4, WS_2_ was identified as the most promising ETL due to its favorable conduction band alignment, low recombination losses, and superior device metrics relative to SnS_2_, In_2_S_3_, and IGZO. Therefore, the subsequent analyses of defect density, interface defect density, thermal response, *J*–*V* and QE characteristics (Sections 3.5 and 3.7) focus primarily on the WS_2_-based configuration, which best represents the optimized device design.

### 3.5. Role of simultaneous variations in defect and acceptor concentrations

**Fig. 6:**
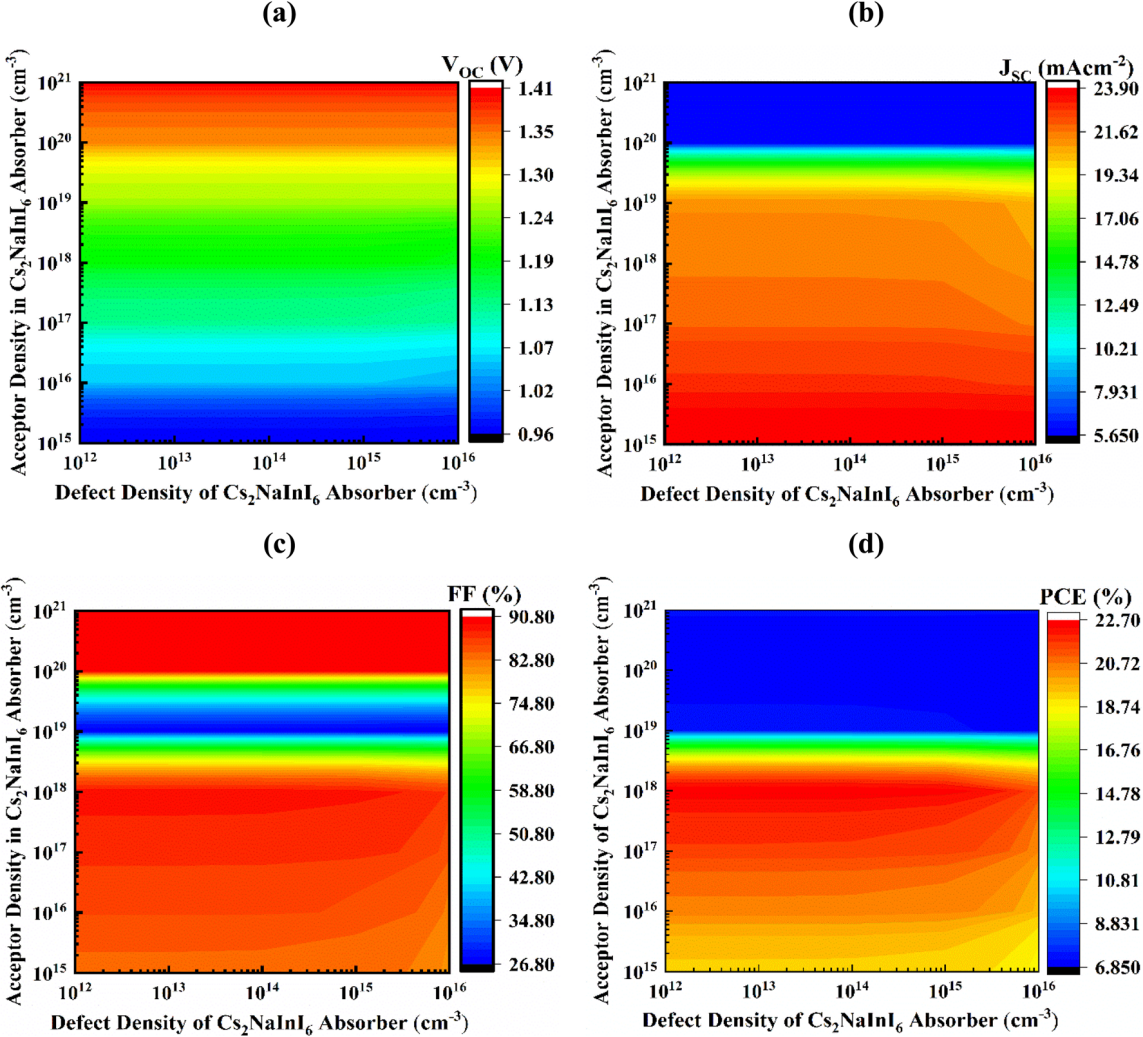
Changes in defect and acceptor density of the Cs_2_NaInI_6_ active layer affect the following output metrics; (a) *V*_OC_ (b) *J*_SC_ (c) FF and (d) PCE.

The device output noticeably declines due to extensive carrier recombination effects, comprising non-radiative recombination, Shockley–Read–Hall, and Auger, produced by larger values of acceptor density (*N*_A_) and defect density (*N*_t_).^47–49^ Elevated recombination of photogenerated carriers markedly reduces cell efficiency.^50–52^ In this study, we investigate how different *N*_A_ and *N*_t_ values within specified boundaries of 10^12^–10^16^ cm^−3^ and 10^15^–10^21^ cm^−3^, respectively, affect output in Cs_2_NaInI_6_ absorber-based PSC. The aim is to evaluate device performance using practical and reliable data for realistic responses, as exhibited in Fig. 6.

Fig. 6(a) vividly demonstrates the impact of *N*_t_ and *N*_A_ on the *V*_OC_ of the ITO/WS_2_/Cs_2_NaInI_6_/Au PSC structure. In this analysis, *N*_A_ is varied over an extensive span ranging from 10^15^ to 10^21^ cm^−3^, even though *N*_t_ spans from 10^12^ to 10^16^ cm^−3^. The *V*_OC_ exhibits a pronounced enhancement, increasing from 0.96 V to a peak value of 1.41 V. An optimized *V*_OC_ of 1.189 V is consistently accomplished once *N*_A_ is maintained at 10^18^ cm^−3^ and *N*_t_ remains below 10^15^ cm^−3^. However, a noticeable degradation occurs when *N*_A_ drops below 10^17^ cm^−3^, causing *V*_OC_ to decline significantly to 0.96 V, underscoring the detrimental effect of inadequate doping. Fig. 6(b) presents the variation in *J*_SC_, which ranges widely from 5.650 mA cm^−2^ to 23.90 mA cm^−2^. The maximum *J*_SC_ of 21.409 mA cm^−2^ is obtained under optimal doping conditions (*N*_A_ = 10^18^ cm^−3^, *N*_t_ < 10^15^ cm^−3^), suggesting enhanced carrier generation and collection efficiency. According to Fig. 6(c), the FF presents a dramatic variation between 26.80% and 90.81%. The optimum FF of 88.92% is observed at *N*_A_ = 10^18^ cm^−3^ and *N*_t_ < 10^14^ cm^−3^, reflecting a highly efficient charge transport mechanism with minimized recombination. Fig. 6(d) further highlights the corresponding variation in PCE, ranging from 6.850% to an impressive maximum of 22.70%. The highest PCE of 22.63% is achieved under the same optimized conditions, reinforcing the strong dependency of overall device performance on finely tuned doping and defect parameters.

### 3.6. Role of interface defect density and temperature on device performance

**Fig. 7:**
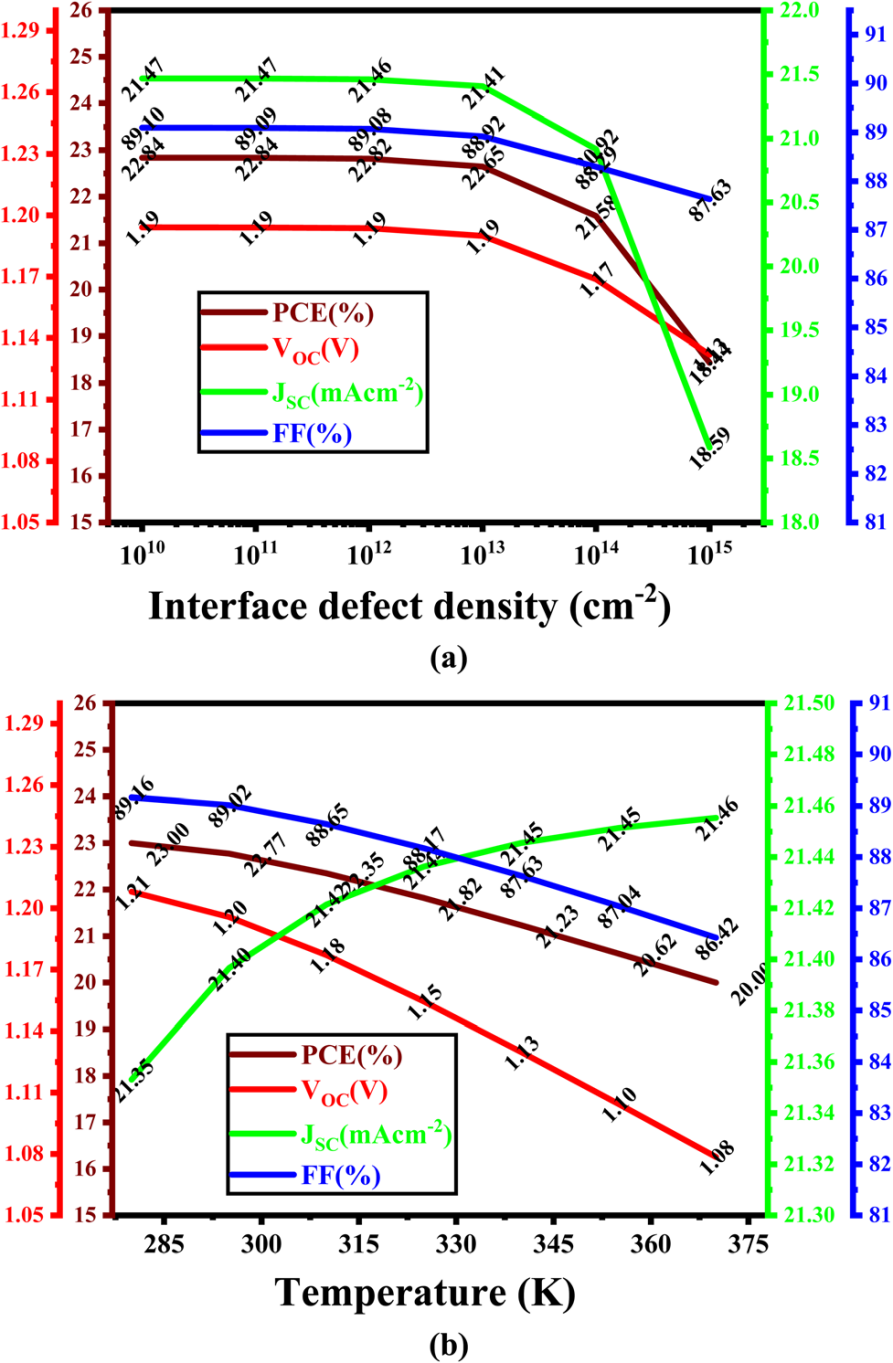
Role of (a) interface defect density and (b) temperature variation on the output of PSCs.

Photogenerated charge carrier populations are critically suppressed by interface trap states that facilitate non-radiative recombination and adversely impact their efficient extraction and transport.^53^ Variations in the defect density at the ETL/absorber interface specifically within the Cs_2_NaInI_6_ double perovskite layer ranging from 10^11^ to 10^15^ cm^−2^, significantly impact the overall photovoltaic output as demonstrated in Fig. 7(a). The interfacial characteristics between the double perovskite active layer and the WS_2_ ETL serves an vital and highly sensitive role in determining device performance.^54–56^ Once the interface defect density surpasses 10^13^ cm^−2^, a pronounced degradation in output is noticed. This performance worsening is primarily linked to the higher availability of trap-assisted recombination pathways, which accelerate charge carrier loss and undermine the built-in electric field, thereby limiting voltage output and carrier collection.^57,58^ As the interface defect density rises, *V*_OC_ drops from 1.19 V down to 1.16 V, with an optimized value of 1.19 V attained at 10^13^ cm^−2^. Similarly, *J*_SC_ declines noticeably from 21.47 mA cm^−2^ to 18.39 mA cm^−2^, with its maximum optimized value of 21.41 mA cm^−2^ also corresponding to the same defect level. Moreover, both FF and PCE follow a downward trajectory, where FF drops from 89.10% to 87.63%, and PCE is reduced from 22.84% to 18.44%. Once the interface defect density surpasses 10^13^ cm^−2^, a pronounced degradation in output is noticed. However, at an interface defect density of 10^13^ cm^−2^, both PCE and *V*_OC_ remain close to their highest simulated values, confirming this point as the optimum condition before significant performance decline occurs. This performance worsening at higher defect levels is primarily linked to the increased availability of trap-assisted recombination pathways,^59^ which accelerate charge carrier loss and undermine the built-in electric field, thereby limiting voltage output and carrier collection. Thus, Fig. 7(a) clearly indicates the optimum interface defect density as 10^13^ cm^−2^.

As described in Fig. 7(b), this study systematically investigates simulated thermal response of the device as the operating temperature varied from 280 K to 370 K, on the output of the proposed PSC architectures. The simulation outcomes clearly reveal that with a progressive rise in temperature, the PCE gradually declines across all device configurations. This efficiency degradation is predominantly attributed to the thermally induced enhancement in reverse saturation current, which intensifies non-radiative carrier recombination and diminishes the overall charge extraction efficiency.^35,60^ Despite this universal trend, Cs_2_NaInI_6_-based PSCs demonstrate remarkable thermal response, particularly in configurations utilizing the WS_2_ ETL. Even under elevated thermal stress, the PCE exhibits only a modest decline, signifying the material's inherent stability and resilience against thermal fluctuations rendering it suitable for potential application in real-world surroundings where operational temperatures can vary substantially.

Furthermore, Fig. 7(b) indicates a distinct downward trend in *V*_OC_ with increasing temperature, most notably in the WS_2_-based configuration. This reduction is fundamentally associated with the temperature-induced narrowing of the semiconductor bandgap, which lowers the quasi-Fermi level splitting and, consequently, the maximum attainable voltage output. In contrast, the *J*_SC_ remains largely unaffected across the entire temperature spectrum. This relative invariance is ascribed to the stability of photogeneration mechanisms, as the incident photon flux and intrinsic absorption characteristics remain substantially constant with temperature, thereby sustaining steady carrier generation. The FF, however, exhibits a consistent decline as the temperature increases. This behavior can be correlated with elevated series resistance and diminished carrier mobility, which collectively impede charge transport and reduce the ideality of the current–voltage (*J*–*V*) behavior. The synergistic lessening in both *V*_OC_ and FF largely contributes to the detected deterioration in PCE. Overall, these observations highlight the significant role of material and interface optimization to boost the thermal stability and maintain the high operational performance of Cs_2_NaInI_6_-based PSCs under diverse environmental conditions.

### 3.7. Analysis of quantum efficiency (QE) and current–voltage (J–V) properties

A comprehensive evaluation of the QE and *J*–*V* behavior is fundamentally critical for elucidating the optoelectronic output of devices. These metrics serve as powerful diagnostic tools, offering quantitative comprehensive grasp of the effectiveness of light absorption, carrier photogeneration, and the electrical response of the solar cell.^40,61,62^ The QE spectrum specifically reflects the proportion of collected charge carriers relative to the incoming photon flux, thereby directly correlating with spectral responsivity. Conversely, the *J*–*V* profile reveals the current output as a function of applied bias, enabling detailed assessment of key parameters.^29,36,63^

**Fig. 8:**
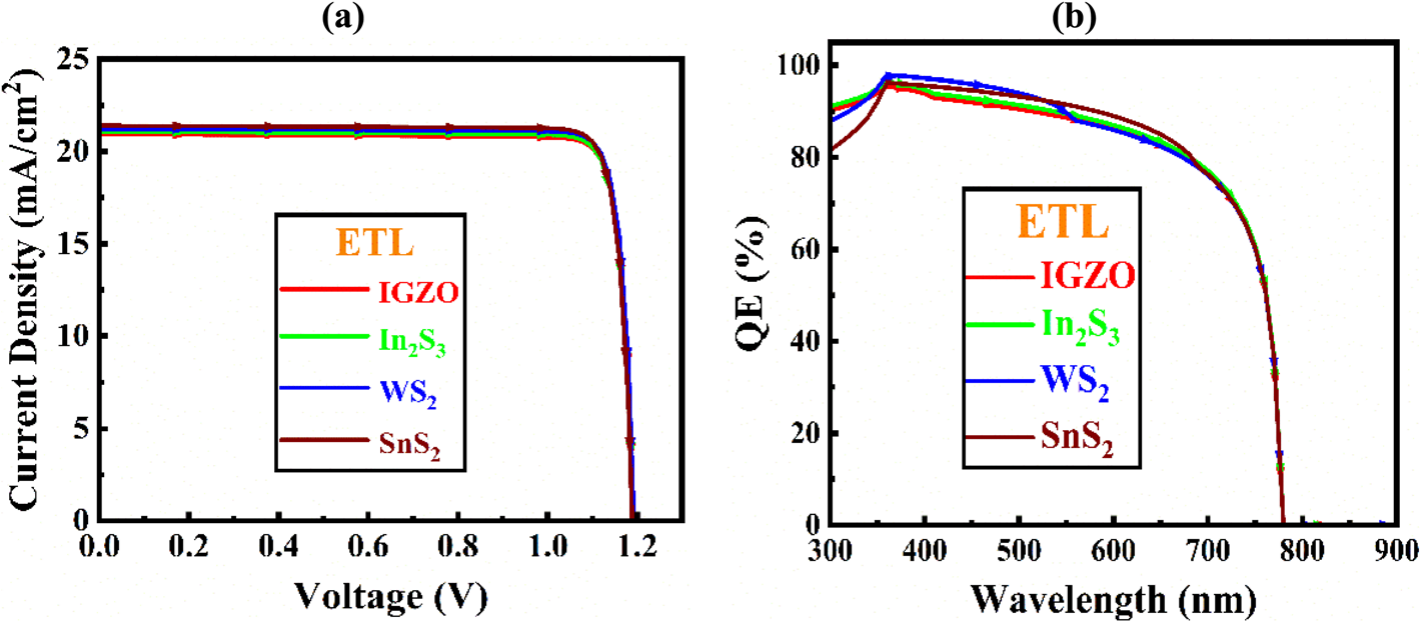
(a) *J*–*V*, (b) QE curve with various ETL of Cs_2_NaInI_6_-based double PSC.

Fig. 8(a) and (b) illustrate the *J*–*V* and QE characteristics for different ETLs. The optimized Al/ITO/WS_2_/Cs_2_NaInI_6_/Au structure was achieved with finely tuned parameters: absorber thickness of 1.2 μm, bulk defect density of 10^14^ cm^−3^, acceptor density of 10^18^ cm^−3^, and interface defect density of 10^13^ cm^−2^. The simulated *J*–*V* curve in Fig. 8(a) shows a *V*_OC_ of 1.189 V and a high *J*_SC_ of 21.409 mA cm^−2^. However, as forward bias exceeds 1.20 V, *J*_SC_ decreases sharply toward zero due to carrier saturation and recombination. Fig. 8(b) presents the QE response, where quantum efficiency rises steadily up to ∼400 nm in the UV-visible region, then gradually declines with increasing wavelength. A sharp drop occurs beyond 700 nm, reaching zero near 790 nm, corresponding to the absorber's bandgap limit. Together, these results highlight the wavelength-dependent absorption and electrical behavior of the optimized device, confirming WS_2_ as an effective ETL and demonstrating the strong efficiency potential of Cs_2_NaInI_6_-based PSCs under practical conditions.

### 3.8. Solar cell performance

Table 3 provides an in-depth comparison of key output metrics for Cs_2_NaInI_6_-based PSCs employing various ETLs. Among the evaluated configurations, the device incorporating WS_2_ as the ETL exhibits the highest PCE of 22.63%, surpassing the others in overall performance. It also achieves a notably high *J*_SC_ of 21.406 mA cm^−2^, a moderately strong FF of 88.92%, and a respectable *V*_OC_ of 1.189 V. Following closely, the SnS_2_-based configuration records a PCE of 22.36%, a *J*_SC_ of 21.234 mA cm^−2^, and an FF of 89.18%, indicating efficient charge transport and collection. The In_2_S_3_-based solar cell demonstrates a competitive performance with a PCE of 22.32%, a *J*_SC_ of 21.083 mA cm^−2^, and an FF of 88.71%. Meanwhile, the IGZO-based device shows the lowest efficiency, with a PCE of 22.23%, a *J*_SC_ of 20.943 mA cm^−2^, and an FF of 88.96%. Overall, the WS_2_ ETL configuration distinctly outperforms the others, offering the most favorable synergy of high efficiency, enhanced current output, and stable voltage, thereby positioning itself as the most promising ETL candidate for high-performance Cs_2_NaInI_6_-based PSCs.

**Table 3:** Comparative Performance Metrics of different double perovskite PSCs Configurations^a^

Structure	*V*_OC_ (V)	*J*_SC_ (mA cm^−2^)	FF (%)	PCE (%)	Ref.
ITO/IGZO/Cs_2_NaInI_6_/Au	1.1932	20.943	88.96	22.23	This work
ITO/In_2_S_3_/Cs_2_NaInI_6_/Au	1.1936	21.083	88.71	22.32	This work
ITO/WS_2_/Cs_2_NaInI_6_/Au	1.189	21.406	88.92	22.65	This work
ITO/SnS_2_/Cs_2_NaInI_6_/Au	1.194	21.234	89.18	22.60	This work
ITO/CuNiO/Cs_2_AgBiBr_6_/C60/BCP/Ag	1.01	3.19	69.2	2.23	E^64^
FTO/SnO_2_/Cs_2_AgBiBr_6_/Cu_2_O/Au	1.511	15.76	69.84	16.63	T^65^
FTO/TiO_2_/Cs_2_AgBiBr_6_/Cu_2_O/Au	1.508	15.71	71.1	16.85	T^65^
FTO/ZnO/Cs_2_AgBiBr_6_/Cu_2_O/Au	1.510	15.75	72.52	16.79	T^65^

a E = Experimental, T = Theoretical.

## 4. Conclusions

This study presents a detailed simulation-based investigation into the performance optimization of Cs_2_NaInI_6_ double perovskite solar cells (PSCs) by carefully selecting and tuning electron transport layers (ETLs). Using SCAPS-1D, four ETL candidates-WS_2_, SnS_2_, In_2_S_3_, and IGZO-were evaluated within a planar ITO/ETL/Cs_2_NaInI_6_/Au architecture. Device performance was systematically analyzed by varying absorber thickness, defect density, doping concentration, and operating temperature to assess their impact on power conversion efficiency (PCE), open-circuit voltage (*V*_OC_), short-circuit current density (*J*_SC_), and fill factor (FF). Among the studied ETLs, WS_2_ delivered the most promising results, achieving a maximum PCE of 22.63%, supported by favorable *V*_OC_, *J*_SC_, and FF values. This superior performance is attributed to its advantageous band alignment, high carrier mobility, and minimized interfacial recombination. The optimized structure, with a 1.2 μm absorber and interface defect density of 10^13^ cm^−2^, also demonstrated strong electronic and thermal stability, underscoring the potential of Cs_2_NaInI_6_ for sustainable photovoltaic applications. Overall, the work highlights the critical influence of ETL engineering on lead-free PSC efficiency and provides simulation-based guidelines for designing environmentally friendly, cost-effective solar devices. However, these stability claims are theoretical, and experimental validation remains essential to confirm long-term device reliability.

## Conflicts of interest

There are no conflicts of interest to declare.

## Data Availability

Data will be available on request.
